# Effect of ultrasonic treatment on enzymes: Decoupling the relation between the ultrasonic driven conformational change and enzyme activity

**DOI:** 10.1016/j.ultsonch.2023.106720

**Published:** 2023-12-09

**Authors:** Bashar Kabawa, Imca Sampers, Katleen Raes

**Affiliations:** Research Unit VEG-i-TEC, Department of Food Technology, Safety and Health, Faculty of Bioscience Engineering, Ghent University, Campus Kortrijk, Sint-Martens-Latemlaan 2B, 8500 Kortrijk, Belgium

**Keywords:** Ultrasound, Energy, Enzyme activity, Enzyme conformation, Pectinase, Cellulase

## Abstract

Considering the growing interest in enzyme-based extraction technology as a safe and eco-friendly extraction technique, along with the relatively high cost associated with enzymatic applications, it became necessary to explore novel strategies aimed to improve enzyme activity. In this study, the impact of ultrasonic treatment on commercial cellulase and pectinase was investigated. As this effect may be influenced by various ultrasonic and enzyme-related parameters, changes in enzyme conformation were explored under optimal and non-optimal enzyme conditions. The intrinsic fluorescence spectrum was utilized as a tool for monitoring these changes. Additionally, the enzyme’s catalytic potential was also assessed under the same conditions. Results indicated that the impact of ultrasonic treatment on enzyme conformation primarily depends on the total ultrasonic energy delivered to the system, rather than other ultrasonic parameters such as power, sample volume, treatment time, or duty cycle. The maximum relative decrease in intrinsic fluorescence intensity of Pectinex® Ultra Clear (PUC) and Pectinex® Ultra SPL (PUS) after ultrasonic treatment was approximately 51% and 55%, respectively, while the decrease induced by thermal denaturation was 25% and 30% respectively. Furthermore, a blue shift in the fluorescence spectrum of both pectinases was observed upon sonication for all process conditions indicating a change in enzyme conformation. However, ultrasonic treatment did not result in a significant change in enzyme activity, suggesting that these conformational adjustments may occur in regions other than the active sites. Moreover, ultrasonicated pectinases and cellulases did not exhibit any improvement in their catalytic potential under either optimal or non-optimal conditions.

## Highlights

• Ultrasound affects enzymes uniquely, often stronger at lower enzyme concentrations.
• Delivered ultrasonic energy is a critical process parameter requiring more attention.
• Sonication induced enzyme conformational change in regions beyond their active sites.
• Certain regions of the enzyme were hidden in its 3D structure upon sonication.

## 1 Introduction

In recent years, enzymes have been increasingly utilized as an effective and eco-friendly tool in many fields of biotechnology. Many studies have already addressed the diverse applications of enzymes such as in bioethanol production and the extraction of valuable bioactive components from plant-based matrices [1], [2]. Unlike conventional extraction methods, enzyme-based extraction is gaining more attention because of its high selectivity and its ability to function in aqueous solutions under mild processing conditions. In this way, the use of organic solvents is avoided, which gives the advantage of eliminating all safety hazards and quality degradation risks related to their presence in the end product since their complete removal is often unfeasible [3]. The basic principle of enzymatic extraction is the hydrolysis and the disruption of the cellular walls of the targeted cells causing the release of not only intracellular compounds but also those bound to the cellular walls, which are often inaccessible in conventional solvent extraction methods [4]. Therefore, enzymes like cellulase, pectinase, xylanase, α-amylase, and others are often used in enzymatic extraction processes. However, the low efficiency and the high costs are usually the main drawbacks of enzymatic extraction methods [2], [5]. One of the approaches that have been recently suggested to enhance enzymatic catalysis is to utilize ultrasound as an assisting technology.

Ultrasound is sound waves at a frequency beyond the range that a human ear can detect [6]. When ultrasonic waves pass through a liquid medium, they create displacement in the liquid particles inducing a succession of compression and refraction in this medium. Strong refractions can increase the distance between contiguous molecules beyond the critical molecular distance which leads to the formation of microbubbles in the liquid. These microbubbles grow as they oscillate in the liquid by compression and refraction cycles until they reach a critical size then they violently implode creating several local effects like high shear force, high temperature, generation of free radicals, and others. This implosion of the microbubbles and the accompanied effects is usually known as the cavitation phenomenon [7]. Most of the studies, dealing with the application of ultrasonic waves on enzymes, have addressed the inactivating effect of ultrasound. It has been reported that ultrasonic treatment can be used to deactivate enzymes responsible for the browning of fruits and vegetables like polyphenol oxidase, peroxidase, and lipoxygenase as well as denaturing pathogens to ensure the safety of food products [8], [9]. However, recent studies proved that ultrasound can also enhance the catalysis of enzymatic reactions if applied in mild conditions [8], [9], [10], [11], [12]. This enhancing effect was mainly explained by coupling more substrate molecules with enzyme active sites through different mechanisms such as improving the mass transfer in the reaction system or acting on the substrate by degrading it, destroying its aggregates, increasing the enzyme-substrate interfacial area in liquid–liquid systems, and decreasing the substrate’s degree of polymerization making it more liable to be attacked by the enzyme [5], [10]. Only a few studies suggested that ultrasonic treatment can enhance enzymatic catalysis by increasing enzyme activity [8], [9], [11], [12], [13]. The most commonly suggested mechanism behind this activity increment is that the cavitation accompanying ultrasonic waves favourably alters the conformation of the enzyme without destroying its three-dimensional structure resulting in the exposure of hidden active sites to the surface of the enzyme and making them accessible to more substrate molecules [13].

However, there is still no consensus on the influential process parameters behind the positive effect of ultrasonic treatment on enzyme activity. This is mainly due to the lack of information about the ultrasonic treatment conditions or the dissensus about how the process parameters should be reported [14]. Many publications have addressed, for instance, the effect of ultrasonic power on enzymes from different aspects. The ultrasonic power in different studies was, however, reported in various units such as power intensity (W/cm^2^) [13], power density (W/ml) [15], or ultrasonic power (W) with no information about the probe diameter [16]. This lack of uniformity in reporting data hinders the reproducibility of the experiment. Assuming that cavitation is the key phenomenon behind this effect, any process parameter that affects the cavitation intensity such as ultrasonic power, treatment temperature, as well as reactor geometry, and the factors affecting the distribution of sound waves should also have an impact on its stimulating ability [14], [17].

The main goal of this study is to investigate the activating effect of ultrasound on enzymes and the mechanism behind this activation. Therefore, the impact of ultrasonic treatment on the activity and conformation of two groups of enzymes (cellulase and pectinase) as representatives of extraction-relevant enzymes was studied under different process parameters including enzyme-related and ultrasound-related parameters. The link between the ultrasound-induced conformational change of the enzyme and the change in its activity was also investigated. Besides, these tests were carried out under optimal and extreme enzyme conditions to follow up on any activation effect under non-optimal conditions or any possible shift in the optimal conditions of the studied enzymes due to ultrasonic treatment.

## 2 Material and methods

### 2.1 Materials

Pectinase from *Aspergillus aculeatus* (Pectinex® Ultra Clear, a blend of pectinases, hemicellulases and arabinases with activity of 8600 PGNU/g) and (Pectinex® Ultra SP-L, a blend of pectinases, hemicellulases and beta-glucanases with activity of 3300 PGNU/g), cellulase from *Trichoderma reesei* (Celluclast® 1.5L, a cellulase aqueous solution with activity of ≥700 units/g) were obtained from Sigma Aldrich (Darmstadt, Germany). Information about the optimal conditions of these enzymes as declared by the producer can be found in Table 1. Microcrystalline cellulose Avicel® PH-101, pectin from citrus peel, 3,5-dinitro salicylic acid, D-(+)-galacturonic acid monohydrate and D-(+)-glucose were purchased from Sigma Aldrich (Darmstadt, Germany). Sodium carbonate anhydrous, citric acid, sodium hydroxide, and L (+)-potassium sodium tartrate tetrahydrate were purchased from VWR Chemicals (Leuven, Belgium), while di-sodium hydrogen phosphate anhydrous, sodium acetate trihydrate, and acetic acid 99–100 % were obtained from Chem-Lab (Zedelgem, Belgium).

**Table 1:** Conditions range of activity of the used enzymes as declared by the producer.

Enzyme	pH range of activity	Temperature range of activity
Pectinex® Ultra Clear	1.8–3.0	25–40 °C
Pectinex® Ultra SP-L	2.8–6.5	15–55 °C
Celluclast® 1.5L	4.0–6.0	50–60 °C

### 2.2 Experimental setup

Enzyme samples were subjected to direct ultrasonic treatments using a probe-type ultrasonic device (Model UP200st by Hielscher, Germany) with a maximum power of 200 Watt and a driving frequency of 26 kHz fitted with a 14 mm diameter (154 mm^2^ surface) probe. Initially, 150 ml samples of 0.1 % w/v enzyme solution were sonicated for 15 min with a US power of 140 W and 50 % duty cycle. Process parameters such as treatment time (5–30 min), duty cycle (50 % and 100 %), sample volume (50–250 ml), and enzyme concentration (0.1 %–0.2 % w/v), were varied one at the time (data not shown). Based on these results, only combinations of parameters were chosen to work further with when the temperature of the treatment did not exceed 40 °C, 55 °C, and 60 °C for PUC, PUS and CEL respectively, as this temperature increase would result in a loss of the enzyme activity. All ultrasonic (US) treatments took place in cylindrical glass reaction vessels with different dimensions and bottom shapes by immersing the probe in the liquid sample at different depths below the liquid surface (1 and 5 cm). The differences between different reaction vessels include differences in internal diameter (6.66, 4.53, and 2.14 cm) and in the bottom shapes (flat/curved). Figure S1 illustrate different bottom shapes of US vessel. Enzyme samples were entitled to the US treatment at an initial temperature equal to the ambient temperature (22 °C) unless stated otherwise. To avoid extreme overheating of the sample caused by the cavitation, the reaction vessel was immersed in an ice bath during the entire US treatment to maintain the temperature under 30 °C. The temperature of the solution was continuously logged.

#### 2.2.1 Sample preparation

Pectinase and cellulase solutions were prepared in two concentrations (0.1 % w/v and 0.2 % w/v) by diluting the commercial enzyme solution in an appropriate amount of a suitable buffer solution at the desired pH. These concentrations correspond to 4165, 3275 and 583 U/L for 0.1 % w/v Pectinex® Ultra Clear (PUC), Pectinex® Ultra SPL (PUS) and Celluclast (CEL) respectively and to 8330, 6550, and 1166 U/L for 0.2 % w/v of these enzymes respectively.

Pectinase samples (PUC) and (PUS) were prepared using a citrate buffer (0.01 M) of different pH (between 2.2 and 5.5), while cellulase samples (CEL) were prepared in acetate buffer (0.05 M, pH 4.8) and citrate buffer (0.05 M, pH between 4 and 7).

#### 2.2.2 Ultrasonic parameters

US treatments were performed at different ultrasonic power (40, 90, and 140 W) which corresponds to 25.97, 58.44, and 90.91 W/cm^2^ respectively and two different duty cycles (50 % and 100 %) where the duty cycle of 50 % represents 10 s of working time followed by 10 s of resting time. The duration of US treatment was also varied (5, 15, and 30 min).

#### 2.2.3 Non-optimal enzyme condition

To evaluate the effect of US treatment on enzymes in extreme conditions, all enzyme samples were examined, in addition to their respective optimal pH condition, under non-optimal pH conditions where the residual activity of the enzymes was proven to be decreased by at least 85 % as compared to optimal pH condition.

Based on the results of Herlet et al., (2017), a pH of 4.8 was chosen as an optimal pH for Celluclast® 1.5L, while pHs 4, 6.5, and 7 were chosen as extreme pHs [18]. For both Pectinex® Ultra Clear and Pectinex® Ultra SP-L, pH 4 was considered as optimal while pH 2.2 and pH 5.5 were chosen as extreme pH conditions [19], [20].

To assess the effect of the extreme incubation temperature, Celluclast® 1.5L was incubated at 65 °C beside its optimal incubation temperature of 50 °C.

#### 2.2.4 Reactor’s geometry

The geometry of the reactor can be understood as the shape of the vessel, the internal dimensions, the shape of the bottom, and the distance between the tip of the probe and the bottom of the reactor. These factors can significantly affect the role of the ultrasound waves if a standing wave field was created [17]. However, adjusting one parameter at a time while fixing other parameters was not possible since these variables are strongly linked to each other. Therefore, a complex setup was designed, as shown in Table 2, to get a comprehensive understanding of the effect of each factor among the shape factors.

**Table 2:** Overview of different geometrical parameters of the ultrasonic vessel.

Studied factor	Code of treatment	volume (ml)	Internal diameter (cm)	Immersed depth of the probe (cm)	Distance under the probe (cm)	Bottom shape^1^
Diameter of the vessel	G2	50	4.53	1	2	Flat
	G3	50	2.14	1	12.35	Curved
Immersed part of the probe	G3	50	2.14	1	12.35	Curved
	G4	50	2.14	5	8.7	Curved
	S	150	6.66	1	3.58	Flat
	G1	150	6.66	5	0.32	Flat
Volume	S	150	6.66	1	3.58	Flat
	V1	250	6.66	1	6.34	Flat

1 An illustration of different bottom shapes of the US vessel is shown in Figure S1.

#### 2.2.5 Enzymatic hydrolysis

To assess the effect of ultrasonic treatment on the hydrolyzing capability of enzymes, both native (untreated) and US-treated enzyme samples were utilized in enzymatic hydrolysis using a suitable substrate (pectin from citrus peel and microcrystalline cellulose Avicel® PH-101 for pectinase and cellulase respectively). The effect of US treatment on enzyme activity was assessed based on a comparison between the amount of reducing groups released by the enzymatic reaction of native and US-treated enzymes after specific incubation time slots.

Pectinase hydrolysis was performed by adding 1 ml of native/US-treated enzyme solution to 4 ml pre-incubated pectin solution (10 g/l) at the optimal temperature of the corresponding used enzyme. The pectin solution was prepared by dissolving pectin from the citrus peel in 0.01 M citrate buffer of the same pH as the tested enzyme, followed by homogenization using an ultraturrax at 16000 rpm for 3 min (T18 digital ultraturrax by IKA, Staufen, Germany). The reaction mixture was then incubated in a water bath at 30 °C for different incubation times (ranging between 2 and 60 min). At the end of the incubation period, the reaction was terminated by adding 180 µl of 1 M NaOH to elevate the pH of the reaction mixture resulting in a complete inactivation of the enzyme. Samples were kept in an ice bath till analysis.

Cellulase hydrolysis was performed by adding 1 ml of native/US treated sample to 4 ml of pre-incubated Avicel suspension (20 g/l) in an acetate or citrate buffer (0.05 M, different pH as described in 2.2.1) at the desired incubation temperature. The reaction mixture was incubated in a water bath at the desired incubation temperature (varying between 50 °C and 65 °C) for a given incubation time (between 5 and 180 min). At the end of the incubation, the reaction was terminated by introducing 50 µl sodium carbonate solution (110 g/l) resulting in an elevation in pH and a complete inactivation of the enzyme, followed by centrifugation (3700 rpm, 4 °C, 10 min) (Z366 K centrifuge, Hermle, Wehingen, Germany). Samples were kept cool till analysis.

### 2.3 Analytical assays

#### 2.3.1 Intrinsic fluorescence analysis

Intrinsic fluorescence spectra of the samples were measured using a spectrofluorometer (RF-5301PC, Shimadzu, Duisburg, Germany) and LabSolutions RF as running software. An excitation wavelength of 280 nm was applied while the emission wavelength range was set between 300 and 450 nm with a data interval of 0.2 nm, medium scanning speed, and spectral bandwidth of 5 nm for both excitation and emission. The corresponding buffer solution was used as a blank while both native and thermal-denatured enzyme samples (same concentration, heated for 10 min at 100 °C) were used as a reference.

#### 2.3.2 Enzyme catalytic capability

The catalytic capability of the enzyme was determined by measuring the concentration of released reducing groups resulting from the hydrolysis of a substrate in a given time in the presence of the studied enzyme. This concentration was determined with the di-nitro salicylic acid method (DNS) as proposed by Miller (1959) with small adjustments [21]. Briefly, 4 ml of DNS reagent was added to 1 ml of the sample, and placed in a boiling water bath for 10 min followed by cooling down to the ambient temperature. The absorbance was then measured at 550 nm using a UV–VIS spectrophotometer (UV-1800, Shimadzu, Duisburg, Germany). Concentrations were calculated based on external calibration curves of the appropriate standard (D-(+)-glucose for cellulase and D-(+)-galacturonic acid monohydrate for pectinase). The limit of detection (LOD) and the limit of quantification (LOQ) for these calibration curves were always maintained below 0.09 and 0.27 mg/ml, for D-(+)-glucose respectively, and below 0.13 and 0.4 mg/ml for D-(+)-galacturonic acid respectively.

#### 2.3.3 Michaelis-Menten kinetics

Michaelis-Menten kinetic parameters (Michaelis-Menten constant K_M_ and the maximum reaction rate V_max_) were determined for (un)treated enzyme samples by measuring the concentration of the enzymatic hydrolysis products (the released reducing groups) at different concentrations of the substrate. K_M_ and V_max_ were calculated based on Lineweaver–Burk plot.

#### 2.3.4 Statistical analysis

All experiments in this study were conducted in triplicate and values were reported as mean ± SD. Statistical analysis was performed using SPSS statistical software version 28.0 (SPSS Inc., Chicago, IL, USA). One-way analysis of variance (ANOVA) and Kruksal-Wallis tests were done to compare the mean values of intrinsic fluorescence intensity among different treatment conditions. Post-hoc Tukey’s test was further used for pairwise comparison. Student t-tests and Mann-Whitney tests (p < 0.05) were conducted to identify any significant differences between the mean values of the two groups. The significance difference was accepted at p < 0.05.

## 3 Results and discussion

### 3.1 Effect of ultrasonic treatment on the enzyme conformation

Enzymes are protein molecules whose catalytic activities depend on their native conformation. Any change in this conformation might lead either to exposing more active sites and making them accessible for the substrate or to hiding already available ones inside its structure and blocking them. Since the activity of the enzyme is strongly related to the number of accessible active sites, altering the protein conformation might lead to a change in its activity. To understand the effect of ultrasonic treatment on enzymes, it is interesting to investigate its effect on the three-dimensional conformation of the enzyme.

Fluorescence spectrometry is a commonly used method to study the alteration in the conformation of a protein based on analyzing the change in the fluorescence spectrum upon a specific manipulation. This fluorescence is attributed to the presence of the aromatic amino acids tryptophan (Trp), tyrosine (Tyr), and phenylalanine (Phe) in the structure of the enzyme and particularly to Trp [13], [22]. The high quantum yield besides its high sensitivity to its local environment qualifies Trp to take priority over Tyr and Phe for the fluorescence measurements [23]. As Trp in a polar environment tends to emit fluorescence light at a higher wavelength as compared to a non-polar environment, the emission spectrum of Trp can be used as a tool to evaluate the amount of Trp residues exposed to the surface of the protein sample upon specific manipulations [23]. In this work, changes in enzyme conformation were investigated mainly by the Trp fluorescence spectrum for all enzymes.

#### 3.1.1 Effect of enzyme-related parameters

**Fig. 1:**
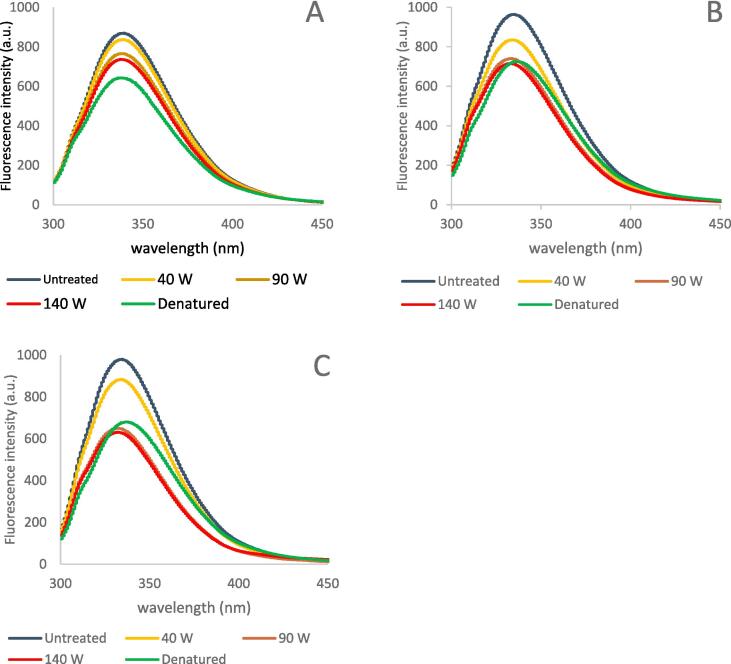
Intrinsic fluorescence spectra of control and US-treated enzymes at different US power 40 W; 90 W; and 140 W, and controlled treatment conditions (volume 150 ml; concentration 0.1 %; treatment time 15 min; duty cycle 50 %). Untreated and thermal denatured enzyme solution (obtained by heating for 10 min at 100 °C) were used as control. A: Celluclast® 1.5 L; B: Pectinex® ultra clear; C: Pectinex® ultra SP-L.

Typical fluorograms for the ultrasonicated studied enzymes under controlled process parameters and different ultrasonic power (0–140 W) are shown in Fig. 1. All ultrasonic treatments induced a decrease in the intrinsic fluorescence intensity of the treated enzymes, independent of the parameter varied. A shift to a different extent in the fluorescence wavelength at the maximum peak towards a shorter wavelength (a blue shift) was observed in sonicated pectinase samples PUC and PUS, while the fluorescence of all sonicated cellulase samples did not show any shift in the wavelength. It can be concluded that the effect of US treatment on the enzyme fluorescence is enzyme-dependent as a US treatment of celluclast® 1.5L (CEL), pectinex® ultraclear (PUC), and pectinex® ultra SPL (PUS) at a given set of process parameters (treatment time 15 min, US power 140 W, duty cycle 50 %, 150 ml sample with a concentration of 0.1 % w/v) reduces the fluorescence intensity by 15 %, 26 %, and 35 % respectively. This enzyme-dependent effect of US treatment on the intrinsic fluorescence intensity is evident since the content of Trp residues and their location in the enzyme structure, as well as the content of quenchers in the enzyme structure varies with the enzyme used. In a previous study on cellulase, a decrease in enzyme fluorescence intensity was also observed upon sonication with no red or blue shift in the optimum fluorescence emission wavelength [22]. It was suggested that these results were due to an increase in the number of Trp molecules on the surface of the cellulase, which indicates that ultrasonic treatment induced molecular unfolding of the enzyme resulting in higher enzyme activity [22]. Similar results were also obtained by Subhedar & Gogate (2014) and by Wang et al. (2012) [2], [13]. On the other hand, the decrease in pectinase fluorescence intensity upon sonication, according to Ma et al. (2020), was attributed to the decrease in the amount of Trp residues on the surface of the enzyme due to the unfolding in its spatial structures. According to the author, this ultrasonic-induced unfolding was beneficial in those cases where the applied ultrasonic treatment was mild (power does not exceed 4.5 W/ml). Unlike our results, a little red shift of 2 nm (from 336 to 338 nm) in the fluorescence spectrum at the maximum peak was observed upon US treatments with high power intensity (higher than 4.5 W/ml). This red shift was attributed to the destruction of the regular folded structure of the enzyme [12].

Table 3 summarizes the effect of different US treatment conditions on the fluorescence intensity of PUC and PUS. When enzymes are present in lower concentrations, the decrease in fluorescence intensity tends to be more pronounced. This suggests that the effect of ultrasonic treatment on the enzyme is negatively related to the concentration of this enzyme. In their study on pectinase, Ma et al. (2020) observed also a relation between the effect of ultrasonic treatment and enzyme concentration. The observed positive effect of ultrasonic treatment on enzyme activity increased as the enzyme concentration decreased. The proposed explanation is that at high enzyme concentrations, the enzyme molecules are more densely distributed as compared to low-concentrated samples, which leads to an attenuated energy transfer process and a lower degree of modification [12].

**Table 3:** The absolute values of fluorescence intensity at the maximum peak for native, thermally denatured, and US-treated pectinases under different process parameters. Results are expressed as a.u. (n = 3).

Treatment	PUC					PUS				
	Untreated	40 W	90 W	140 W	denatured	Untreated	40 W	90 W	140 W	denatured
Initial treatment (S)	972.0 ± 7.0 ^AB,a^	836.0 ± 14.7	741.0 ± 7.2	715.0 ± 4.4 ^A,b^	725.3 ± 25.7	971.7 ± 10.4 ^A,a^	884.7 ± 22.0	650.3 ± 9.1	631.7 ± 22.0 ^A,b^	681.0 ± 30.8
Duty cycle 100 %	972.0 ± 7.0 ^AB,a^	766.7 ± 6.1	651.3 ± 17.0	638.3 ± 10.5^b^	725.3 ± 25.7	971.7 ± 10.4 ^A,a^			621.3 ± 13.9^b^	681.0 ± 30.8
Treatment time 5 min	972.0 ± 7.0 ^AB,a^	907.7 ± 8.7	862.7 ± 7.1	838.7 ± 7.1^b^	725.3 ± 25.7	971.7 ± 10.4 ^A,a^			879.3 ± 12.7^b^	681.0 ± 30.8
Treatment time 30 min	972.0 ± 7.0 ^AB,a^			710.7 ± 9.9^b^	725.3 ± 25.7	971.7 ± 10.4 ^A,a^			628.3 ± 9.2^b^	681.0 ± 30.8
Enzyme concentration 0.2 % (w/v)	918.3 ± 9.7 ^A,a^	892.0 ± 4.4	832.3 ± 9.3	816.0 ± 21.7^B,b^	730.3 ± 16.2	991.3 ± 5.5 ^A,a^			823.3 ± 6.1 ^A,b^	661.0 ± 13.0
V1 (250 ml)	972.0 ± 7.0 ^AB,a^	886.7 ± 13.9	836.3 ± 34.0	787.3 ± 5.9^b^	725.3 ± 25.7	971.7 ± 10.4 ^A,a^	973.7 ± 22.6	881.0 ± 11.4	850.3 ± 24.8^b^	681.0 ± 30.8
G1	972.0 ± 7.0 ^AB,a^			681.7 ± 2.9^b^	725.3 ± 25.7	971.7 ± 10.4 ^A,a^			590.7 ± 2.3^b^	681.0 ± 30.8
G2	972.0 ± 7.0 ^AB,a^			603.3 ± 45.0^b^	725.3 ± 25.7	971.7 ± 10.4 ^A,a^			501.3 ± 21.6^b^	681.0 ± 30.8
G3	972.0 ± 7.0 ^AB,a^			575,7 ± 38.2^b^	725.3 ± 25.7	971.7 ± 10.4 ^A,a^			491.3 ± 29.8^b^	681.0 ± 30.8
G4	972.0 ± 7.0 ^AB,a^			475.0 ± 35.0^b^	725.3 ± 25.7	971.7 ± 10.4 ^A,a^			437.3 ± 5.9^b^	681.0 ± 30.8
pH 2.2	936.0 ± 2.6 ^AB,a^			711.3 ± 7.5 ^A,b^	654.3 ± 7.5	902.0 ± 16.4^B,a^			554.3 ± 33.3^B,b^	562.3 ± 33.3
pH 5.5	1015.7 ± 0.6^B,a^			845.0 ± 5.3^B,b^	771.0 ± 28.6	983.3 ± 32.0 ^A,a^			722.3 ± 18.5^C,b^	756.3 ± 51.3

Initial treatment (S) was carried out under the following settings: (US probe immersion depth: 1 cm; US power: 140 W; duty cycle: 50 %; treatment time: 15 min; enzyme concentration: 0.1 % (w/v); pH 4; sample volume: 150 ml; in a cylindrical US vessel with a flat bottom and an internal diameter of 6.66 cm). G1-G4 are US treatments of samples in different reactors geometry.

Significant differences (p < 0.05) are denoted by different letters, with capital letters representing comparisons across columns and small letters representing comparisons within rows.

#### 3.1.2 Effect of ultrasonic parameters

**Fig. 2:**
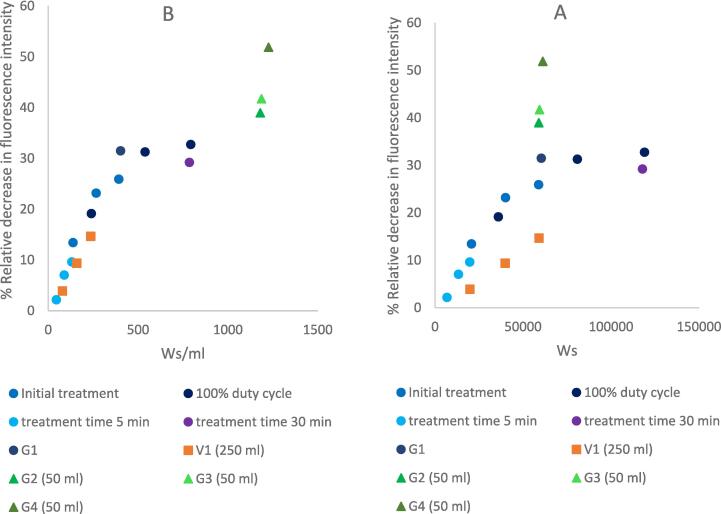
Relationship between the total US energy applied to enzyme solution and the decrease in its fluorescence intensity. (A) represents the relative decrease in fluorescence intensity in the function of the US energy for different sample volumes; (B) represents the relative decrease in fluorescence intensity in the function of the US energy density (energy per volume unit of all sample volumes). Initial treatment conditions are (15 min treatment time, 50 % duty cycle, 150 ml sample volume, enzyme concentration 0.1 % w/v), G1–G4 and V1 are US treated enzyme samples in different reactor geometries and the same initial treatment conditions unless stated otherwise.

Fig. 2 illustrates the relation between the total sonotrode output ultrasonic energy (different treatment times, duty cycles, and US power) and the decrease in the intrinsic fluorescence intensity of PUC. There is a clear linear relationship between the relative decrease in the fluorescence intensity and the increase in the applied US energy up to 60,000 Ws for a sample volume of 150 ml. Further increase in US energy beyond this value led to a plateau in fluorescence intensity. Moreover, integrating the results of the US treatment of different sample volumes under the same conditions fit in this linear model, indicating that the sample volume does not have an enormous impact if the geometry is kept the same. The outliers in G2, G3 and G4 can be attributed to the geometry of the treatment vessel and the length of the immersed part of the probe. Indeed, the effect of US treatment on enzymes depends more on the total ultrasonic energy density (energy per volume unit) of the system than on the US power, treatment time, or duty cycle. This approach of determining the influential process parameters on enzymes has not yet been implemented, to the best of our knowledge. This is due to the difficulty in obtaining the total delivered energy, as not all ultrasonic devices provide this option. The majority of the studies address the US power and the treatment time as the influential process parameters that affect enzymes. Tran et al. (2018) suggested that 75 s of US treatment with a power of 25 W/ml are the optimal process parameters to improve the catalytic activity of α-amylase by 47 %. A study on the changes in the secondary structure of the enzyme upon sonication was conducted to support their findings [11]. As far as we know, only one other study suggested that the US duty cycle also affected the activity of β-D-glucosidase, as the extensive cavitation present at higher duty cycles can affect the structure of the enzyme [24].

The decrease in the fluorescence intensity of a protein is mainly attributed to the displacement of the Trp residues in the protein structure to places in molecular contact with quenchers. These quenchers could be either an amino acid in the peptide backbone (such as histidine or lysine) or a water-soluble quencher (collisional quencher) present in the water phase such as molecular oxygen. When a change in the enzyme conformation occurs in which Trp residues exposes to the water phase, water-soluble quenchers that come in contact with Trp residues are mainly the reason behind the decrease in the fluorescence intensity of this Trp molecule [23]. The gradual decrease in the effect of ultrasound on the fluorescence intensity with increasing energy levels may be explained by a partial saturation of all quenchers with Trp molecules limiting their quenching capacity. Besides, this might also indicate that the cavitation effect is only able to modify the enzyme conformation to a limited extent beyond which the enzyme structure cannot be modified further.

On the other hand, it can also be seen in Fig. 1C that some ultrasonic treatments induced a further decrease in fluorescence intensity of both PUC and PUS as compared to those caused by thermal denaturation. As widely known, thermal denaturation causes a complete breaking down of the three-dimensional structure of the enzyme, bringing all Trp residues to the surface and in contact with the water phase. This breakage in the enzyme structure can be observed as a decrease in the fluorescence intensity accompanied by a shift in the emission wavelength at the maximum peak to a longer wavelength (red shift). This denaturation-induced red shift is often remarked in proteins that do not contain exposed Trp residues [23]. The decrease in fluorescence intensity in sonicated enzymes beyond the level induced by thermal denaturation indicates that a modification in the enzyme conformation caused by US treatment could have hidden some regions of the enzyme inside the three-dimensional structure and put Trp residues in molecular contact with some quenchers inside the enzyme structure. This suggestion can be supported by the observed blue shift in the fluorescence spectra of PUS and PUC at the maximum peak in almost all ultrasonic treatments, as seen in Table 4. The only study, in which a shift in the fluorescence spectra upon ultrasonic treatment was observed, was also on pectinase. However, this shift was, contrary to our findings, only in samples sonicated at high power levels (US power higher than 180 W) and toward longer wavelengths (from 336 to 338 nm). This red shift was attributed to the destruction of the regular folded structure of the enzyme by which the catalytic function of the enzyme was barely maintained [12].

**Table 4:** The emission wavelength at the maximum peak of native and US-treated pectinases under different process parameters (n = 3).

Treatment	PUC					PUS				
	Untreated	40 W	90 W	140 W	denatured	Untreated	40 W	90 W	140 W	denatured
Initial treatment (S)	335	333	334	333	336	335	334	332	332	337
Duty cycle 100 %	335	333	333	332	336	335			334	337
Treatment time 5 min	335	334	334	333	336	335			333	337
Treatment time 30 min	335			333	336	335			333	337
Enzyme concentration 0.2 % w/v	334	334	334	334	337	334			335	337
V1 (250 ml)	335	334	334	333	336	335	335	333	333	337
G1	335			333	336	335			332	337
G2	335			332	335	335			332	337
G3	335			331	336	335			332	337
G4	335			329	336	335			330	337
pH 2.2	334			333	338	334			332	339
pH 5.5	335			334	338	334			333	337

Initial treatment (S) was carried out under the following settings: (US probe immersion depth: 1 cm; US power: 140 W; duty cycle: 50 %; treatment time: 15 min; enzyme concentration: 0.1 % (w/v); pH 4; sample volume: 150 ml; in a cylindrical US vessel with a flat bottom and an internal diameter of 6.66 cm). G1-G4 are US treatments of samples in different reactors geometry..

#### 3.1.3 Effect of reactor geometry

As listed in Table 3, all ultrasonic treatments in which the probe was deeply immersed in the liquid (5 cm below the liquid surface) show a higher decrease in fluorescence intensity as compared to their counterparts in which the probe was only 1 cm deep immersed, regardless the shape of the reactor’s bottom. As for 150 ml samples, the decrease in fluorescence intensity of sonicated PUC and PUS in the case of deep immersion of the probe (G1) was about 5 % and 3 % more as compared to the initial treatment (S), respectively. Similar results were obtained when the sample volumes were adjusted to 50 ml and sonicated in a curved-bottom reactor using two different probe immersion depths (G3 and G4). Specifically, a more than 11 % and 6 % reduction in fluorescence intensity was observed for PUC and PUS, respectively, when the probe was deeply immersed, compared to the same treatment with the probe immersed only 1 cm below the liquid surface. This difference is less likely to be related to the shape of the vessel’s bottom, as only a slight difference in fluorescence intensity can be seen when sonicating in two vessels with different bottom shapes when other process parameters are fixed (G2 and G3). This difference in the relative decrease of fluorescence intensity might be ascribed to the way the ultrasonic waves propagate in the system in case of different immersion depths. It has not been established, however, whether the distance below the probe has also an impact on the decrease in fluorescence intensity upon sonication. This factor is difficult to be followed up on as it is related to other geometry-related factors, such as the diameter and the immersion depth of the probe. Very few studies addressed vessel geometry as a potential factor that might affect the efficiency of ultrasound applications with enzymes. A previous study has established that applying a stainless steel reflector on the bottom of the ultrasonic reactor during sonication can intensify the effect of sonication, leading to significant retrogradation of commercial cellulase [17].

#### 3.1.4 Effect of non-optimal enzyme conditions

Extreme pH values are widely known to affect the enzyme conformation causing a permanent or reversible denaturation [9]. It is also suggested that the beneficial effect of US treatment might be more pronounced in conditions far from optimal conditions [25]. Therefore, the effect of US treatment on PUC and PUS was also investigated at pH conditions where the catalytic activity is proven to be considerably decreased [19], [26]. It can be noticed from Table 3 that the application of US treatment at the initial parameters (treatment time 15 min, US power 140 W, sample volume 150 ml) at optimal pH conditions induced a decrease in fluorescence intensity by about 26.4 % and 35 % for both PUC and PUS respectively. By varying the pH to more acidic conditions (pH 2.2), no considerable change in the effect of ultrasound on the fluorescence intensity was observed (24 % and 38 % decrease at pH 2.2 as compared to 26 % and 35 % at pH 4 for PUC and PUS respectively). However, the effect of US treatment on the fluorescence intensity became less pronounced when the pH was increased toward more neutral conditions at pH 5.5 (17 % and 26 % decrease in fluorescence intensity at pH 5.5 compared to 26 % and 35 % for PUC and PUS respectively). Based on the observed results, it can be concluded that the impact of the US treatment on the intrinsic fluorescence intensity of the enzyme is dependent on the pH level, with a greater decrease observed at lower pH values and a weaker decrease at higher pH values approaching neutrality. This pH-related decrease in fluorescence intensity can be attributed to the formation of free radicals as a result of the cavitation since the formation of these radicals is more pronounced in acidic environments compared to neutral or basic environments [27]. On the other hand, all US treatments result in a shift in wavelength to a consistent range between 329 and 333 nm, regardless of the pH of the environment. This shift suggests that US treatments at various pH values cause a similar amount of Trp residues to be buried within the enzyme structure.

### 3.2 Effect of ultrasonic treatment on the enzyme activity

**Fig. 3:**
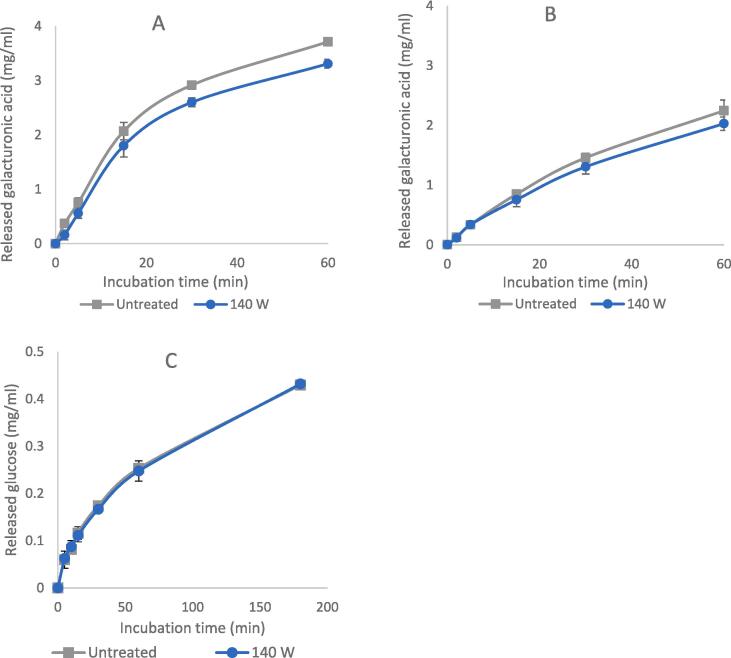
Effect of initial US treatment on the activity of pectinase PUC (A), pectinase PUS (B), and cellulase CEL (C) expressed in mg released corresponding reducing group by means of enzymatic hydrolysis (n = 3).

To evaluate the effect of US treatment on the enzyme activity, the amount of released reducing groups through enzymatic hydrolysis of native and sonicated enzymes for fixed incubation timeslots were compared. Fig. 3 depicts a comparison in catalytic potential between native and ultrasonicated enzymes at different ultrasonic power. The ultrasonic treatment of all studied enzymes did not lead to a change in the activity. Only a slight decrease in the released galacturonic acid can be observed after 30 min of hydrolysis by sonicated PUC and PUS. This might indicate a decrease in enzyme stability due to sonication which led to a slight decrease in the catalytic potential after a specific incubation period. These results are partly consistent with those of Dalagnol et al. (2017), as they also did not detect any change in pectinase activity resulting from ultrasonic treatment. However, they observed an increase in cellulase activity by 40 % due to a pre-sonication of the enzyme for 30 min [9]. On the other hand, it has been reported that ultrasonic treatment was able to intensify pectinase activity by 68 %, 21 % and 19 % when pectinase solutions of different concentrations (0.1, 1.0, and 10.0 g/l respectively) were subjected to 4.5 W/ml of ultrasonic waves [12].

**Fig. 4:**
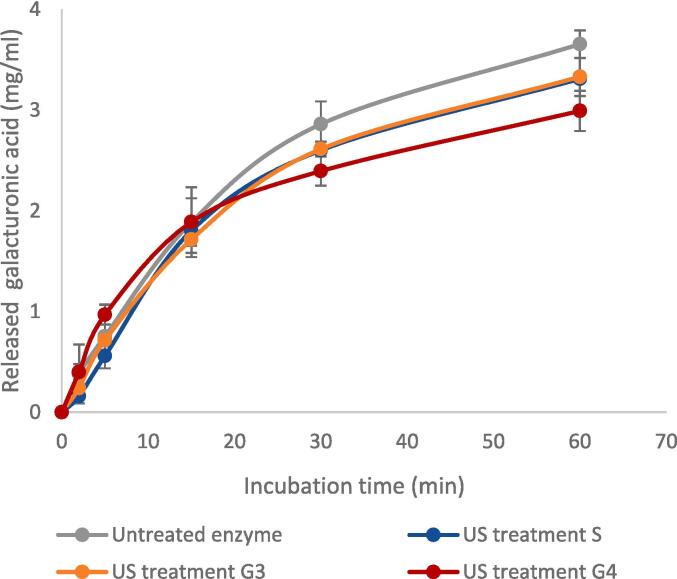
Effect of US treatment under different reactor’s geometry on the activity of pectinase PUC (n = 3).

It may be worthwhile to compare these results to those of the conformation study. Although US treatments induced changes in structural conformation of both pectinases (PUC and PUS) as mentioned in 3.1.1, changes in enzyme activity upon these treatments could barely be found. Moreover, enzyme samples that showed fluorescence intensity decrease by sonication beyond that caused by thermal denaturation, such as US treatments G3 and G4 (Table 3), could even maintain their full activity except for a slight decrease in the quantity of reducing groups from 30 min onwards of incubation, as seen in Fig. 4. These findings confirm that the decrease in fluorescence intensity observed as a result of ultrasonic treatments does not represent a destructive unfolding of the three-dimensional structure of the enzyme, as opposed to the changes observed after thermal denaturation. They might also indicate that US treatment did not work directly on active sites, since no actual change in enzyme activity was observed, but induced some conformational changes and possible unfolding in regions other than those on which the active sites are located.

**Fig. 5:**
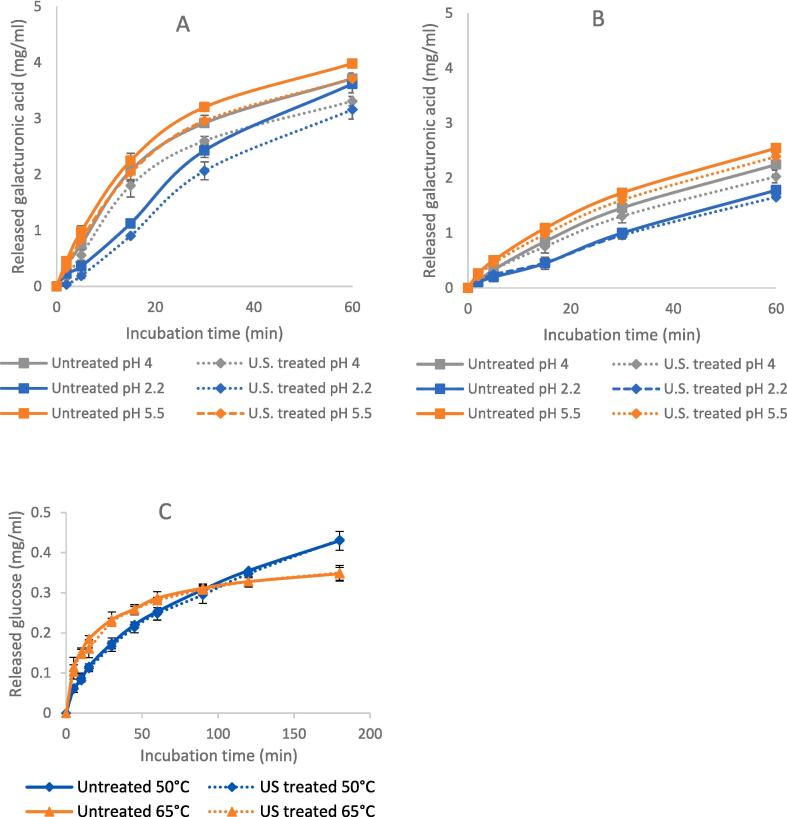
Effect of US treatment on the activity of cellulase CEL, pectinase PUC and pectinase PUS under extreme conditions expressed in mg released reducing group through enzymatic hydrolysis. (A) Pectinase PUC under a range of pH; (B) Pectinase PUS under a range of pH; (C) CEL under non-optimal temperature conditions. All pectinase US treatments were held at the same process parameters: (treatment time 15 min; sample volume 150 ml; US power 140 W; duty cycle 50 %; enzyme concentration 0.1 % w/v; US process parameters for cellulase are: (treatment time 5 min; sample volume 250 ml; US power 120 W; duty cycle 100 %; enzyme concentration 0.2 % w/v).

Given the established literature that ultrasonic-induced enzyme activation is often observed under non-optimal conditions [25], this study also evaluated the catalytic potential of cellulase, PUC and PUS under extreme pH conditions (4, 6.5 and 7 for cellulase; 2.2 and 5.5 for PUC; 2.2 and 4 for PUS) and extreme temperature conditions (65 °C for CEL) (Fig. 5). Interestingly, the ultrasonic impact on PUC and PUS activity was not affected by changes in enzyme pH or incubation temperature. A slight decrease in the quantity of released galacturonic acid could barely be observed after 30 min of hydrolysis. The same trend was also observed for cellulase, as no pronounced difference in the quantity of released reducing sugars was detected after 180 min of hydrolysis using sonicated and native cellulase. In a previous study on invertase, it was reported that ultrasonication of the enzymatic reaction system resulted in an increased degree of conversion at temperatures below the optimal temperature for the enzyme. However, pre-treatment of the enzyme with ultrasonication did not increase its activity across all conditions [25]. In another study by Dalagnol et al. (2017), the activity of pectinase, xylanase, and cellulase was evaluated within a pH range of 3 to 7 in both ultrasonic and mechanical stirring water baths. The results indicated that enzyme activity was consistently higher in the ultrasonic bath as compared to the mechanical stirring bath. However, a relative decrease in xylanase activity under extreme pH conditions (pH 7) was observed in the ultrasonic bath, but to a lesser extent than observed in the mechanical stirring bath. Similar observations were made for cellulase, with a relative decrease in activity under extreme pHs (3 and 7) in the ultrasonic bath being less pronounced than in the mechanical stirring bath. Furthermore, the study also revealed that the enhancement of xylanase and cellulase activity under higher temperature conditions was more pronounced when the reaction was performed in an ultrasonic bath compared to a mechanically stirred bath [9]. If our assumption regarding the dependency of the ultrasonic effect on the total ultrasonic energy delivered to the system was correct, it would be challenging to reconcile the differing results reported in the literature, as most publications do not provide this information in their data.

However, when comparing the results of the catalytic potential of PUC and PUS under non-optimal enzyme conditions with those obtained from evaluating the changes in enzyme conformation in 3.1.4 and Table 3, it can be observed that a significant impact of ultrasonic treatment on enzyme conformation did not translate to a corresponding impact on the catalytic potential of these enzymes. This observation supports the earlier hypothesis that ultrasonic treatments primarily induce conformational changes in regions of the enzyme other than those containing the active sites.

### 3.3 Effect of ultrasonic treatment on the Michaelis-Menten kinetics

To understand the effect of US on the enzyme activity, Michaelis-Menten kinetic parameters were calculated by using the linear transformation of the Michaelis-Menten curve. Table 5 lists the Michaelis constant K_M_ and the maximum reaction rate V_max_ of the native and ultrasonic-treated PUC and PUS as obtained from Lineweaver-Burk plots. The results indicated that the reciprocal of the initial reaction rate (1/V) versus the reciprocal of the substrate concentration (1/S) had a good relationship for both enzymes, as the R^2^ for both native and ultrasonically treated enzymes were 0.993 and 0.997 for PUC and 0.994 and 0.973 for PUS, respectively. Only a slight change in kinetic parameters for PUC is detected after ultrasonication. However, ultrasonication increased the K_M_ of PUS from 2.90 g.L^-1^ to 4.68 g.L^-1^ proposing a lower enzyme-substrate affinity, while the value of V_max_ showed only a slight increase after sonication. This increase in K_M_ values for the US-treated PUS may be attributed to ultrasound-induced conformational changes in the enzyme's binding sites, leading to a decreased affinity between the enzyme and its substrate. This could explain the slight reduction in galacturonic acid release observed after 30 min of hydrolysis by the US-treated PUS.

**Table 5:** Michaelis-Menten kinetic parameters of both native and US-treated pectinases.

	Native enzyme		Ultrasonically treated enzyme	
	K_M_ (g.L^-1^)	V_max_ (g.L^-1^.min^−1^)	K_M_ (g.L^-1^)	V_max_ (g.L^-1^.min^−1^)
Pectinex® Ultra Clear (PUC)	6.482	0.266	5.784	0.236
Pectinex® Ultra SPL (PUS)	2.897	0.097	4.684	0.104

## 4 Conclusion

In this study, the effect of ultrasonic treatment on commercial cellulase (Celluclast® 1.5L) and pectinase (Pectinex® Ultra Clear and Pectinex® Ultra SPL) was investigated. It was established that the effect of ultrasound is enzyme-dependent. The cavitation phenomenon caused by the applied ultrasonic field can significantly impact enzyme conformation. This change in enzyme conformation indicates a hiding of certain regions within the enzyme's three-dimensional structure, rather than a complete breakdown and unfolding of the enzyme's three-dimensional structure as observed in the case of thermal denaturation. The results of the activity measurements support these findings and suggest that conformational changes take place in regions of the enzyme other than the active sites, as no significant change in enzyme activity was observed upon ultrasonication. Furthermore, while changes in enzyme conformation are influenced by various US process parameters including treatment time, power, duty cycle, and sample volume. It has been established that these changes are more accurately represented when expressed in terms of total delivered energy per unit volume. This parameter encompasses other process parameters such as treatment time, duty cycle, ultrasonic power, and sample volume. Additionally, the results suggest that the effect of ultrasonic waves on enzyme conformation depends on the pH of the environment, as a more intense effect is observed at low pH conditions compared to more neutral pH conditions. However, no effect of ultrasonic treatment on enzyme activity could be observed under non-optimal enzyme conditions (pH and temperature).

## CRediT authorship contribution statement

**Bashar Kabawa:** Conceptualization, Methodology, Formal analysis, Investigation, Writing – original draft, Writing – review & editing, Visualization. **Imca Sampers:** Conceptualization, Resources, Writing – review & editing. **Katleen Raes:** Conceptualization, Methodology, Resources, Writing – review & editing, Supervision, Funding acquisition.

## Declaration of Competing Interest

The authors declare that they have no known competing financial interests or personal relationships that could have appeared to influence the work reported in this paper.
