# Supplementary material for: Effect of ultrasonic treatment on enzymes: Decoupling the relation between the ultrasonic driven conformational change and enzyme activity
Source: Ultrason Sonochem. 2023 Dec 9;101:106720. doi: 10.1016/j.ultsonch.2023.106720 (PMC10733687; doi:10.1016/j.ultsonch.2023.106720)
Supplement: Supplementary data 1 [file mmc1.docx]

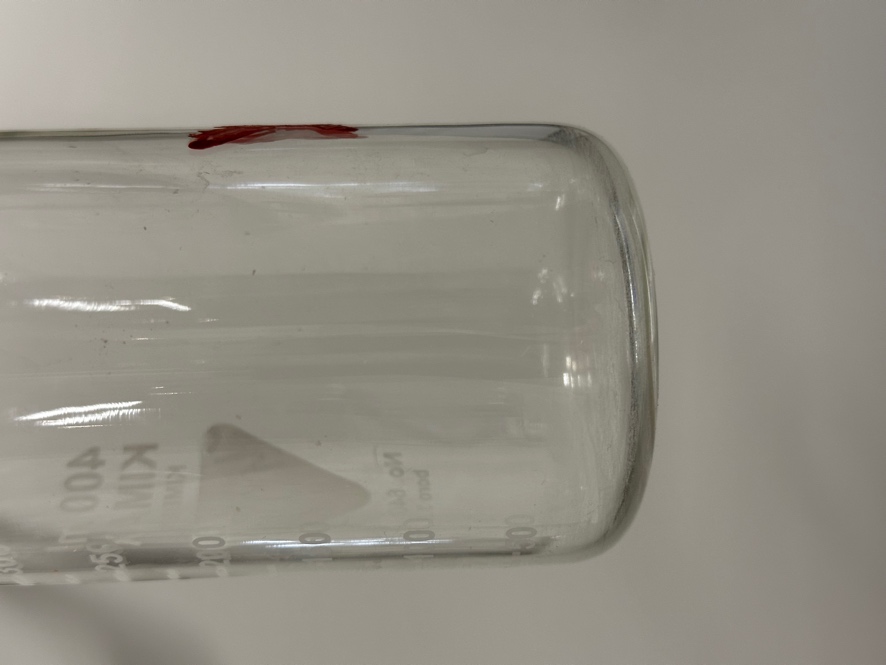

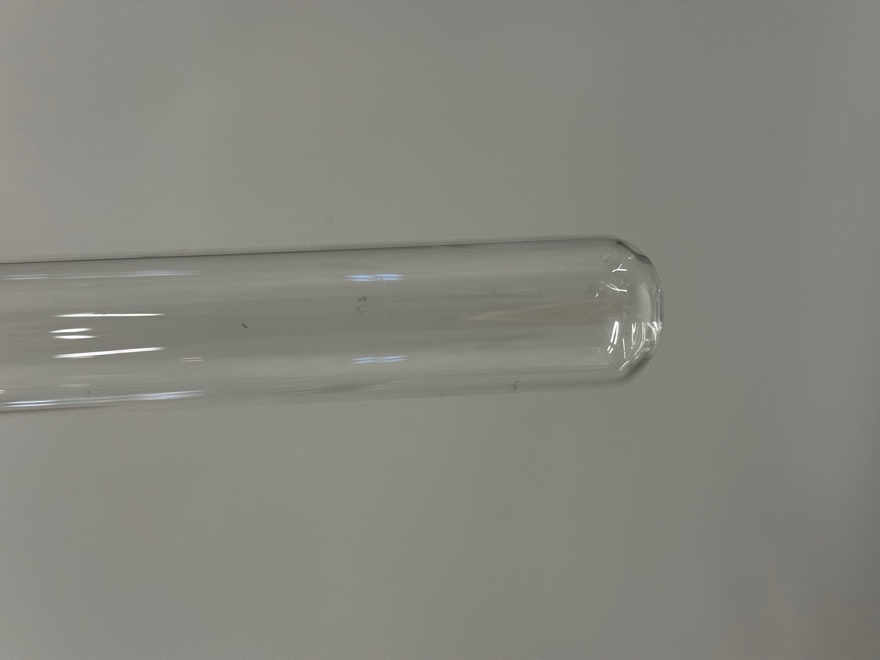


B

A

B.Kabawa et al. An illustration of different bottom shapes of the ultrasonic vessel: (A) flat bottom; (B) curved bottom, Figure S1
